# Percutaneous Bioelectric Current Stimulation in the Treatment of Chronic Achilles Tendinopathy: Protocol for a Double-Blind, Placebo-Controlled Randomized Multicenter Trial

**DOI:** 10.2196/40894

**Published:** 2022-11-11

**Authors:** Philipp Schröder, Albrecht Molsberger, Attyla Drabik, Matthias Karst, Harry Merk

**Affiliations:** 1 Columbus Health Products GmbH Duesseldorf Germany; 2 Department of Orthopedics Ruhr-University Bochum Düsseldorf Germany; 3 Drabik Clinical Trial Support Münster Germany; 4 Department of Anesthesiology Pain Clinic Hannover Medical School Hannover Germany; 5 Meoclinic GmbH Berlin Germany

**Keywords:** Achilles tendinopathy, pain, PBCS, conservative treatment

## Abstract

**Background:**

The consensus for the optimal treatment strategy for chronic Achilles tendinopathy (AT) is still debated and treatment options are limited. This results in a significant medical need for more effective treatment options.

**Objective:**

The aim of this study is to investigate the therapeutic effects of percutaneous bioelectric current stimulation (PBCS) on AT.

**Methods:**

A multicenter, randomized, double-blind, placebo-controlled clinical trial will be conducted. A total of 72 participants with chronic (ie, >3 months) midpoint AT will be randomized and receive four PBCS sessions—either verum or placebo—over 3 weeks. Both groups will complete daily Achilles tendon loading exercises in addition to the intervention. Evaluation sessions will be completed at baseline and during the intervention (weeks 0-3). Self-reported outcome measures will be completed at follow-up at weeks 4, 12, 26, and 52. The primary outcomes are the Victorian Institute of Sports Assessment–Achilles questionnaire scores and statistical evaluation of intraindividual differences between baseline and 12-week evaluations after initial treatment of verum therapy compared to control. Secondary outcomes will assess Pain Disability Index scores; average pain, using the 11-point Numeric Rating Scale; return to sports; and use of emergency medication.

**Results:**

The study began in May 2021. As of October 2022, we randomized 66 out of 72 participants. We anticipate completing recruitment by the end of 2022 and completing primary data analysis by March 2023.

**Conclusions:**

The study will evaluate the effects of PBCS on pain, physical function, and clinical outcomes.

**Trial Registration:**

German Clinical Trials Register DRKS00017293; https://tinyurl.com/mvz7s98k

**International Registered Report Identifier (IRRID):**

DERR1-10.2196/40894

## Introduction

Achilles tendinopathy (AT) is a painful overuse injury [1] and is particularly common in athletes. The prevalence of AT among runners is estimated to be between 6.2% and 9.5% [2], with the highest prevalence (83%) among middle-distance runners [3].

AT is caused by increased stress on the Achilles tendon, especially when running long distances in hilly terrain [4]. People older than 35 years are particularly susceptible [4] to AT. Most commonly, the pain and thickening of the Achilles tendon occur in the midportion of the Achilles tendon, especially 2 to 6 cm distal to the insertion of the Achilles tendon to the heel (ie, the calcaneus) [5].

Achilles tendons of patients with AT show advanced degeneration and changes in the arrangement of collagen fibers. Furthermore, there is ventral sprouting of new blood vessels and associated nerve endings [6]. These are considered the main cause of pain, although there are microscopic changes without clinical symptoms as well as clinical symptoms without microscopic changes. In this respect, the cause of the pain is not fully understood. Since these microscopic changes are considered as degenerative rather than inflammatory, the term tendinopathy is used instead of tendinitis [7].

The leading symptom of AT is load-dependent pain in the course of the Achilles tendon, usually associated with swelling. In the acute form, increasing pain occurs at the Achilles tendon a few centimeters above the heel over a period of a few days. The pain can be temporarily relieved by immobilization [8]. Manual examination of the Achilles tendon reveals that it is swollen, reddened, and hardened. Acute AT often progresses to a chronic course [9]. Symptoms may persist for months to years. The pain remains at about the same intensity with any type of exercise or sports activity, but increases when running uphill or climbing stairs. After resting or in the morning, the pain is especially severe because of stiffness and loss of elasticity of the Achilles tendon. Rupture or partial rupture of the tendon is a frequent complication.

AT is a clinical diagnosis based on localized tendon pain and swelling and pain with activities. Imaging, such as sonography or magnetic resonance imaging, can be used to assess tendon morphology and pathologic conditions.

Therapeutic options for AT are limited [1,10]. Current conservative treatment strategies involve the topical or oral application of nonsteroidal anti-inflammatory drugs (NSAIDs), loading exercises (ie, eccentric-concentric loading), cooling, taping, and exercise rehabilitation. Nonsurgical therapies are also used, including acupuncture, focused shock wave treatment, and electrotherapies (ie, different forms of transcutaneous electrical nerve stimulation [TENS]) [11-13]. In cases of long-standing symptoms that cannot be managed conservatively, surgical splitting of the tendon lengthwise and excision of necrotic tissue can be attempted [14]. However, studies show that the long-term results of surgical therapy do not differ from those of conservative therapy and that operative treatments have a higher risk of other complications [15]. The best evidence for treating AT is available for loading exercises (ie, eccentric-concentric loading) [16]; for all other forms of therapy, evidence is contradictory or anecdotal. Overall, it must be concluded that the optimal treatment strategies for chronic AT are still being debated and that treatments are protracted and mostly unsatisfactory. Given the high prevalence of unsatisfactory treatment options, there is a significant medical need for more effective treatment options.

Percutaneous bioelectric current stimulation (PBCS) treatment is a form of microinvasive electrotherapy different from the aforementioned TENS-like approaches. PBCS mimics and increases physiological electric fields to modulate local tissue inflammation and to trigger the regeneration of nerves, muscles, ligaments, and tendons.

In this study, PBCS using the DC Stimulator Mobile (original equipment manufacturer [OEM] version; Axomera-Molsberger), which has already shown promising results in the treatment of ligaments, tendons, and muscle tissue [17], will be compared to a control treatment without electrical output.

## Methods

### Study Design

This study is a randomized, double-blind, placebo-controlled multicenter trial.

**Figure 1:**
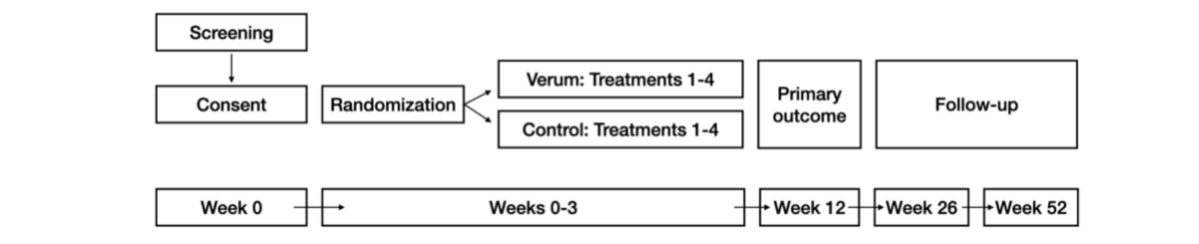
Flowchart of the study process. The verum group will receive PBCS treatment with electrical output as described above. The control group will receive PBCS treatment as described above with no electrical output: the current and voltage will be equal to zero. PBCS: percutaneous bioelectric current stimulation.

Participants will be randomized to either the verum group or the control group. In addition to the intervention, participants in both randomization groups will receive the same baseline therapy in the form of tendon-loading exercise. Each participant will receive four treatment sessions within 3 weeks of inclusion. Evaluation sessions, where primary outcome data are collected, will be conducted at baseline (ie, inclusion) and at week 4. Self-reported follow-ups will be conducted at weeks 4, 12, 26, and 52 (Figure 1).

In this study, the medical device DC Stimulator Mobile (OEM version; Axomera-Molsberger) developed for PBCS treatment will be tested with regard to its technical and medical performance and safety. The aim is the statistical evaluation of therapeutic effects of PBCS with electrical output (verum) in participants with achillodynia compared to PBCS treatment without electrical output (control) as an additional benefit (ie, “add-on”) to an evidence-based standard therapy. This trial was retrospectively registered at German Clinical Trials Register (DRKS00017293) in February 2022.

### Recruitment

Study participants will be recruited directly at the trial sites. Recruitment will be supported using posters, mass emails, and direct approach of potential participants at the study centers. Prior to inclusion in the study, each participant will be informed by the investigator about the nature, significance, risks, and scope of the clinical trial, as well as about the right to terminate participation in the study at any time without incurring any negative effects. Generally understandable information documents will be handed out.

Participants must be given a reflection period of at least 24 hours to decide whether to participate in the study. In addition, they must be given the opportunity to clarify any unanswered questions beforehand. Study-specific investigations to verify the inclusion and exclusion criteria will be performed after the participant has given legally effective consent to the study. Only participants who meet all inclusion criteria will be included and randomized into the SMART-TRIAL electronic data capture (EDC) platform. Participants who do not meet the eligibility criteria will be excluded.

The inclusion criteria will be as follows: achillodynia diagnosis confirmed by a consulting doctor, pain in the Achilles tendon approximately 2 to 7 cm from calcaneus insertion, pain intensity of at least 4 on the 11-point Numeric Rating Scale (NRS) on at least one day in the last 7 days before the start of treatment, aged 18 to 65 years, Achilles tendon pain for 3 or more months, adequate communication skills, and participant must be able to recognize the nature, significance, and scope of the clinical trial and to direct his or her will accordingly.

The exclusion criteria will be as follows: needle phobia; previous PBCS treatment or eccentric training, as specified in this study, of the affected Achilles tendon; Achilles tendon pain for more than 2 years; BMI greater than 30 (obesity grade I); in women, pregnancy; pain intensity of 9 points or higher on the 11-point NRS on any day in the last 7 days before the start of treatment; inability to technically perform the Victorian Institute of Sports Assessment (VISA); pending disability pension application; pacemaker; history of surgery on the Achilles tendon; cortisone injection to the Achilles tendon in the last 3 months; chronic pain of other etiology with ongoing pain management; anticoagulant therapy within 7 days before start of treatment—taking acetylsalicylic acid up to 100 mg/day is not an exclusion criterion; taking opiates within the last 4 weeks before start of treatment; history of taking fluoroquinolone, antibiotics, or statins within the past 6 months; analgesic, drug, or opiate dependence; alcoholism; local infection; type 2 diabetes; renal disease requiring dialysis; autoimmune disease; vascular disease; peripheral neuropathy; other nerve compression syndromes of the lower extremities; radiculitis; rheumatoid arthritis; Reiter syndrome; and other medical reasons as determined by the study physician.

### Randomization

Structural equality of both study arms will be achieved by randomization. Randomization will be performed using the randomization module of the SMART-TRIAL EDC platform. Allocation to the two study arms will be in the form of permuted blocks of variable length, stratified by study site in an allocation ratio of 1 to 1.

### Interventions and Blinding

Four mandatory PBCS treatments will be performed within 3 weeks after inclusion. There must be at least 2 days between treatments.

**Figure 2:**
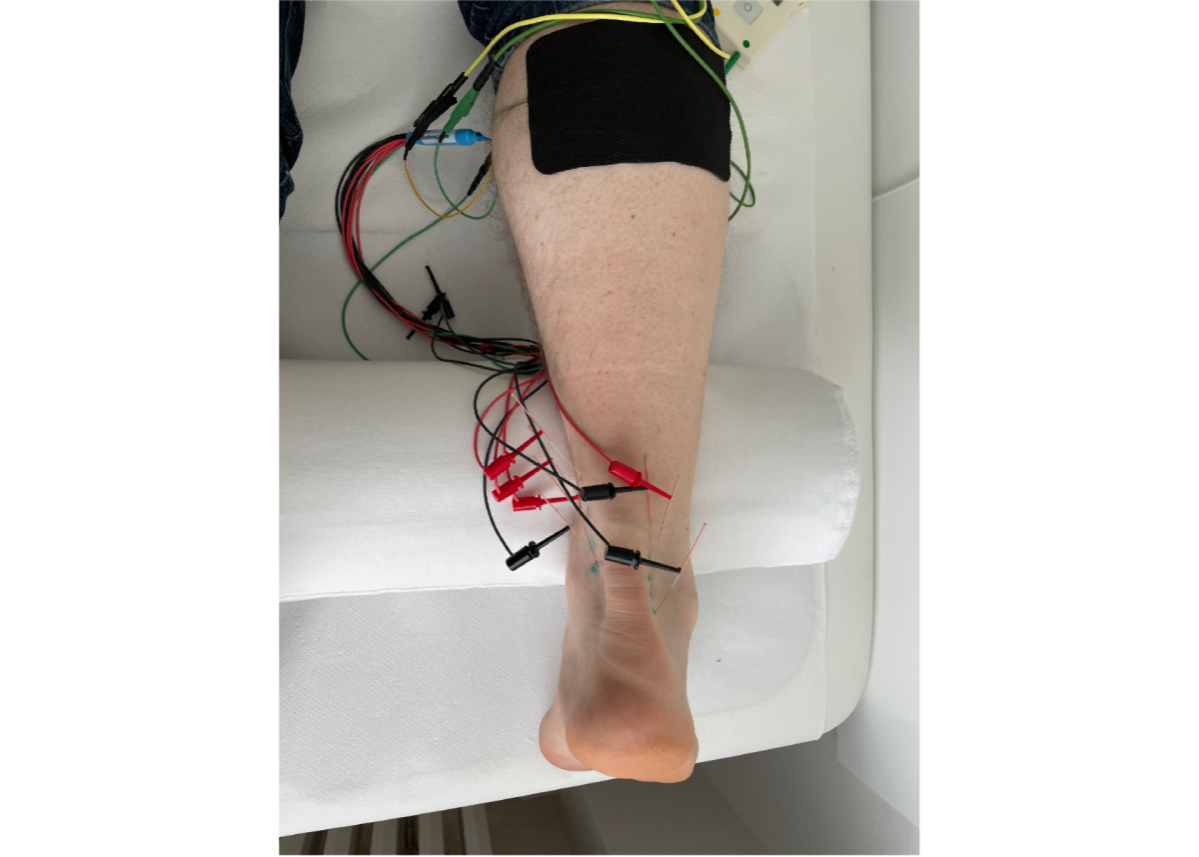
Intervention with PBCS therapy. PBCS: percutaneous bioelectric current stimulation.

Depending on the extent of the painful area, 2 to 8 stainless steel needles (0.20-0.3 mm) will be used. Each needle will be inserted into the affected (ie, paratendinous) tissue until the participant feels the tip of the needle exactly at the painful area. The needle will then be withdrawn a few millimeters so that the tip of the needle is as close as possible to the painful area. The needle probes will be individually connected to the PBCS stimulator via clips and flexible cables. PBCS direct current stimulations will then be performed over 30 minutes according to the indication-specific stimulation protocol; electrical signals will be of the order of 140 mV/ mm, and the drive current will be approximately 20 to 50 μA/cm (Figure 2).

Double-blinding is ensured by the identical design of the treatment hardware. One of the connection cables, however, is technically modified in such a way that no current will be conducted, thus generating no electrical stimulation. The difference in the connection cables will not be detectable by either investigator or participant. The success of blinding will be additionally measured using a blinding question after the first treatment: the participant will be asked whether he or she thinks that he or she has been treated with or without electrical stimulation.

### Outcome Measures

#### Primary Outcome

The VISA–Achilles questionnaire (VISA-A) score [18] will be used as the primary outcome measure. Intraindividual differences between values at baseline and values 12 weeks after initial treatment with verum therapy compared to control will be evaluated. The VISA-A questionnaire is an index of the severity of a clinically diagnosed condition (ie, AT). It contains eight questions on three domains of pain, function, and activity. Scores are summed to a total with a maximum of 100. In this study, the German adaptation (VISA-A-G) will be used. The VISA-A-G questionnaire was tested for reliability, validity, and internal consistency [19].

#### Secondary Outcomes

Secondary outcome measures will be as follows:

1. Statistical evaluation of intraindividual differences in the VISA-A score and the Pain Disability Index (PDI), including all subscales, between values at baseline and weeks 4, 12, 26, and 52 after verum therapy compared to control.
2. Pain on exertion after standing on one leg for 30 seconds, using the 11-point NRS: statistical evaluation of intraindividual differences between values at the beginning of treatment and before each therapy session and at weeks 4, 12, 26, and 52 after verum therapy compared to control.
3. Average pain on average exertion in the last week, using the 11-point NRS: statistical evaluation of intraindividual differences between values at therapy start and before each therapy session and at weeks 4, 12, 26, and 52 after verum therapy compared to control.
4. Return to exercise: statistical evaluation of intraindividual differences between values at baseline and at weeks 4, 12, 26, and 52 after verum therapy compared to control.
5. Treatment response: statistical evaluation of verum therapy compared to control.
6. Use of emergency medication (eg, the NSAID acetaminophen) within 1 week: statistical evaluation of intraindividual differences between values at baseline and at weeks 4, 12, 26, and 52 after verum therapy compared to control.

### Safety Evaluation Criteria

Safety evaluation criteria will be as follows:

1. Listing by month of treatment of adverse events (AEs), adverse reactions (ARs), serious AEs (SAEs), and serious ARs (SARs) stratified by organ classes and events: total sum, total AEs and ARs, total SAEs and SARs, total AEs and SAEs, and total ARs and SARs will be formed.
2. Total listing of AEs, ARs, SAEs, and SARs, including suspected unexpected SARs, stratified by participant and organ classes and events: formed as the total sum, sum of AEs and ARs, sum of SAEs and SARs, sum of AEs and SAEs, and sum of ARs and SARs.
3. Total listing of AEs, ARs, SAEs, and SARs stratified by organ class and severity (ie, intensity): formed as the total, total AEs and ARs, total SAEs and SARs, total AEs and SAEs, and total ARs and SARs.
4. Listing of reasons for study exclusion (ie, violation of inclusion and exclusion criteria) with the duration of study participation to date.
5. Calculation of the probability of occurrence of the respective number of AEs that occurred.

### Sample Size

The sample size is calculated on the basis of the primary outcome, taking into account a clinically relevant effect size between verum and control.

In this study, the treatment effect is considered clinically relevant if the effect size between study arms is at least Δ/σ = 0.75.

To demonstrate a significant treatment effect in the primary statistical analysis with 80% power using a 2-sided Wilcoxon-Mann-Whitney test at the *α*=.05 level, a sample size of 62 participants in total is needed, with 31 participants per intervention group.

The allocation ratio is 1 (verum) to 1 (control). Assuming a dropout rate of 15%, a total of 72 participants should be included in the study.

### Statistical Analysis

Statistical evaluation will generally be carried out using descriptive methods in the form of frequency tables and statistical parameters, such as means, SDs, and quantiles. As graphical procedures, bar charts will be created for qualitative data, and box-and-whisker plots will be created for quantitative data. In addition, inferential statistical analyses will be performed using appropriate significance tests and CIs. Missing values will not be replaced.

The primary statistical evaluation will be performed with a 2-sided Wilcoxon-Mann-Whitney test at the global significance level of *α*=.05; the results will be interpreted in a confirmatory sense.

The evaluation of the secondary evaluation criteria of efficacy will be performed with adequate 2-sided tests. Here, local levels (local level *α*=.05) will be controlled instead of the global significance level, and no adjustment will be made for multiple testing. *P* values of the secondary evaluation criteria will be interpreted descriptively only.

The safety evaluation criteria will be evaluated exploratively. In the exploratory evaluation of the safety criteria, adjustment for multiple testing would be counterproductive and will, therefore, not be performed.

For the primary target criterion, the following 2-sided test problem will be set up:

H_0_: *d*=0 versus H_1_: *d*≠0

where *d* indicates the effect size between intervention groups.

The null hypothesis is as follows: in the statistical evaluation of intraindividual differences between scores at start of treatment and at week 12, there will be no difference in the VISA-A score for symptom assessment between verum and control.

The research hypothesis is as follows: in the statistical evaluation of intraindividual differences between values at start of treatment and at week 12, there will be a difference in the VISA-A score for the assessment of symptomatology between verum and control.

For the secondary outcome of efficacy, corresponding 2-sided test problems will be set up and solved.

The evaluation of the primary and secondary outcomes will be performed according to the intention-to-treat (ITT) principle. The respective collective includes all participants included in the study, regardless of possible protocol violations (eg, study discontinuations or premature discontinuation of intervention). In addition to the ITT analyses, sensitivity analyses will be performed according to the per-protocol principle. Relevant protocol violations leading to exclusion from the per-protocol collective will be defined in the statistical analysis plan. The statistical analysis plan will be prepared in a blinded review without knowledge of the target criteria.

The safety evaluation criteria will be evaluated using the as-treated principle. That is, all participants who participated in the study and received at least one dose of the study intervention (ie, safety collective) will be included.

Subgroup analysis will only be done exploratively, as previous studies have shown that, for example, gender appears not to be a risk factor for AT [20].

### Ethics Approval

The first positive ethics vote was issued on March 27, 2020, by the North Rhine Medical Association (reference No. U1111-1233-2760). Based on this, further positive ethics votes were subsequently issued for the other federal states in Germany where patients are recruited. Informed consent will be given before enrollment. This trial will be conducted in accordance with the ethical guidelines of the Declaration of Helsinki.

## Results

Onboarding of trial sites started at the end of 2020. Enrollment of the first participant occurred on May 28, 2021. As of October 2022, we randomized 66 out of 72 participants. We anticipate completing recruitment by the end of 2022 and completing primary data analysis by March 2023.

## Discussion

### Overview

Evidence-based therapies for AT are rare, leaving practitioners and patients with limited treatment options [21]. Against this background, PBCS has the potential to become one of the few effective therapies available. Therefore, this clinical trial will compare the therapeutic effects of verum versus control PBCS on AT. We aim to show that PBCS could improve function, pain, and other clinical outcomes by measuring differences between VISA-A, NRS, and PDI scores. Although many cases of AT can heal spontaneously, chronic courses, where the symptoms persist for months or years, are frequent. There is no clear evidence on whether AT that includes partial tendon rupture needs to be treated surgically [15]. Surgical treatment of acute Achilles tendon ruptures has been shown to reduce the risk of rerupture compared with nonoperative treatment. However, rerupture rates were low, and differences between treatment groups were small [15,22]. Surgery and injections are associated with various risks, mostly attributable to increased risk of infection [23,24]. Limited therapeutic options for treating AT result in a significant medical need for research and development of effective conservative treatments. With the PBCS treatment, we aim to advance nonsurgical treatment options for practitioners. Up to now, there have been no other comparable randomized controlled trial studies available on microinvasive direct current stimulation for the treatment of AT.

Unlike electroacupuncture or TENS therapy, the PBCS stimulation current is so low—in the range of a few microamperes instead of milliamperes—that it cannot be perceived by the patient. Consequently, the patient cannot reliably distinguish between a verum and a placebo stimulation. This makes it possible, unlike with TENS and electroacupuncture, to actually blind the verum and placebo electrostimulation to the patient and the therapist. In addition, the success of the blinding is checked by a blinding question within the study.

### Limitations

Insertion of needle electrodes is mandatory for the application of PBCS. Prior studies have shown that no placebo control for needle insertion is available and that needling itself can have clinical effects [25]. For this reason, therapeutic effects of needling may influence the results independently of the study intervention. In the verum and placebo groups, needle electrodes are positioned and inserted in an identical manner. The practitioner locates the particularly pain-sensitive points in the area of the Achilles tendon and advances the needle to the painful point. This procedure corresponds to dry needling or professional acupuncture at locus dolendi points; it has been shown that this kind of needling can produce therapeutic effects in pain disorders [26,27]. Therefore, the mere insertion of the needle probes, independent of electrical stimulation, may raise the success rate in both verum and control groups. In light of this consideration, we will additionally examine whether the therapeutic effect of electrical PBCS exceeds that of pure needle insertion. We are convinced that this will further strengthen the clinical relevance of the study.

### Conclusion

This study will evaluate the effects of PBCS on pain, physical function, and clinical outcomes in AT patients. Given the refractory nature and limited therapeutic options for AT, we aim to advance evidence-based nonsurgical care for patients and practitioners.

## Appendix

Multimedia Appendix 1 CONSORT-eHEALTH checklist (V 1.6.1).

## Abbreviations

AE: adverse event
AR: adverse reaction
AT: Achilles tendinopathy
EDC: electronic data capture
ITT: intention-to-treat
NRS: Numeric Rating Scale
NSAID: nonsteroidal anti-inflammatory drug
OEM: original equipment manufacturer
PBCS: percutaneous bioelectric current stimulation
PDI: Pain Disability Index
SAE: serious adverse event
SAR: serious adverse reaction
TENS: transcutaneous electrical nerve stimulation
VISA: Victorian Institute of Sports Assessment
VISA-A: Victorian Institute of Sports Assessment–Achilles questionnaire
VISA-A-G: German adaptation of the Victorian Institute of Sports Assessment–Achilles questionnaire

## Data Availability

All data sets supporting the conclusions of the study (ie, all raw data) will be made available in publicly available repositories by the first author upon publication of the final results for analysis and reanalysis.

## References

[1] Silbernagel K, Hanlon S, Sprague A (2020). Current clinical concepts: Conservative management of Achilles tendinopathy. J Athl Train.

[2] Lopes AD, Hespanhol LC, Yeung SS, Costa LOP (2012). What are the main running-related musculoskeletal injuries?. Sports Med.

[3] Janssen I, van der Worp H, Hensing S, Zwerver J (2018). Investigating Achilles and patellar tendinopathy prevalence in elite athletics. Res Sports Med.

[4] Sobhani S, Dekker R, Postema K, Dijkstra PU (2013). Epidemiology of ankle and foot overuse injuries in sports: A systematic review. Scand J Med Sci Sports.

[5] van Sterkenburg MN, van Dijk CN (2011). Mid-portion Achilles tendinopathy: Why painful? An evidence-based philosophy. Knee Surg Sports Traumatol Arthrosc.

[6] Ackermann P, Salo P, Hart D (2016). Tendon innervation. Adv Exp Med Biol.

[7] Kader D, Saxena A, Movin T, Maffulli N (2002). Achilles tendinopathy: Some aspects of basic science and clinical management. Br J Sports Med.

[8] Li H, Hua Y (2016). Achilles tendinopathy: Current concepts about the basic science and clinical treatments. Biomed Res Int.

[9] Alfredson H, Lorentzon R (2000). Chronic Achilles tendinosis: Recommendations for treatment and prevention. Sports Med.

[10] Scott A, Huisman E, Khan K (2011). Conservative treatment of chronic Achilles tendinopathy. CMAJ.

[11] Ackermann PW, Phisitkul P, Pearce CJ (2018). Treatment of Achilles tendinopathy: State of the art. J ISAKOS.

[12] Imaeda M, Hojo T, Kitakoji H, Tanaka K, Itoi M, Inoue M (2018). Effect of electroacupuncture stimulation on long-term recovery following Achilles tendon rupture in a rat model. Acupunct Med.

[13] Zhang B, Zhong L, Xu S, Jiang H, Shen J (2013). Acupuncture for chronic Achilles tendnopathy: A randomized controlled study. Chin J Integr Med.

[14] Baltes TPA, Zwiers R, Wiegerinck JI, van Dijk CN (2017). Surgical treatment for midportion Achilles tendinopathy: A systematic review. Knee Surg Sports Traumatol Arthrosc.

[15] Ochen Y, Beks RB, van Heijl M, Hietbrink F, Leenen LPH, van der Velde D, Heng M, van der Meijden O, Groenwold RHH, Houwert RM (2019). Operative treatment versus nonoperative treatment of Achilles tendon ruptures: Systematic review and meta-analysis. BMJ.

[16] Malliaras P, Barton CJ, Reeves ND, Langberg H (2013). Achilles and patellar tendinopathy loading programmes: A systematic review comparing clinical outcomes and identifying potential mechanisms for effectiveness. Sports Med.

[17] Molsberger A, McCaig C (2018). Percutaneous direct current stimulation – A new electroceutical solution for severe neurological pain and soft tissue injuries. Med Devices.

[18] Robinson J, Cook JL, Purdam C, Visentini PJ, Ross J, Maffulli N, Taunton JE, Khan KM, Victorian Institute of Sport Tendon Study Group (2001). The VISA-A questionnaire: A valid and reliable index of the clinical severity of Achilles tendinopathy. Br J Sports Med.

[19] Lohrer H, Nauck T (2010). [Validation of the VISA-A-G questionnaire for German-speaking patients suffering from Haglund's disease] [Article in German]. Sportverletz Sportschaden.

[20] Alrashidi Y, Alrabai HM, Alsayed H, Valderrabano V (2015). Achilles tendon in sport. Sports Orthop Traumatol.

[21] van der Vlist AC, Winters M, Weir A, Ardern CL, Welton NJ, Caldwell DM, Verhaar JAN, de Vos R (2021). Which treatment is most effective for patients with Achilles tendinopathy? A living systematic review with network meta-analysis of 29 randomised controlled trials. Br J Sports Med.

[22] She G, Teng Q, Li J, Zheng X, Chen L, Hou H (2021). Comparing surgical and conservative treatment on Achilles tendon rupture: A comprehensive meta-analysis of RCTs. Front Surg.

[23] Holland C, Jaeger L, Smentkowski U, Weber B, Otto C (2012). Septic and aseptic complications of corticosteroid injections: An assessment of 278 cases reviewed by expert commissions and mediation boards from 2005 to 2009. Dtsch Arztebl Int.

[24] Lohrer H, David S, Nauck T (2016). Surgical treatment for achilles tendinopathy - A systematic review. BMC Musculoskelet Disord.

[25] Birch S, Alraek T, Kim KH, Lee MS, Leung S, Hu H (2016). Placebo-controlled trials in acupuncture: Problems and solutions. Evidence-based Research Methods for Chinese Medicine.

[26] Krey D, Borchers J, McCamey K (2015). Tendon needling for treatment of tendinopathy: A systematic review. Phys Sportsmed.

[27] Vickers AJ, Vertosick EA, Lewith G, MacPherson H, Foster NE, Sherman KJ, Irnich D, Witt CM, Linde K, Acupuncture Trialists' Collaboration (2018). Acupuncture for chronic pain: Update of an individual patient data meta-analysis. J Pain.

